# Qualitative Research Methods in Chronic Disease: Introduction and Opportunities to Promote Health Equity

**DOI:** 10.1146/annurev-publhealth-012420-105104

**Published:** 2021-12-22

**Authors:** Rachel C. Shelton, Morgan M. Philbin, Shoba Ramanadhan

**Affiliations:** 1 Department of Sociomedical Sciences, Mailman School of Public Health, Columbia University, New York, NY, USA; 2 Department of Social and Behavioral Sciences, Harvard T.H. Chan School of Public Health, Harvard University, Boston, Massachusetts, USA

**Keywords:** chronic disease, qualitative inquiry frameworks, qualitative methods, qualitative analysis, qualitative research, health equity, health disparities

## Abstract

Public health research that addresses chronic disease has historically underutilized and undervalued qualitative methods. This has limited the field’s ability to advance (*a*) a more in-depth understanding of the factors and processes that shape health behaviors, (*b*) contextualized explanations of interventions’ impacts (e.g., why and how something did or did not work for recipients and systems), and (*c*) opportunities for building and testing theories. We introduce frameworks and methodological approaches common to qualitative research, discuss how and when to apply them in order to advance health equity, and highlight relevant strengths and challenges. We provide an overview of data collection, sampling, and analysis for qualitative research, and we describe research questions that can be addressed by applying qualitative methods across the continuum of chronic disease research. Finally, we offer recommendations to promote the strategic application of rigorous qualitative methods, with an emphasis on priority areas to enhance health equity across the evidence generation continuum.

## QUALITATIVE RESEARCH IS CRITICAL TO UNDERSTAND AND ADDRESS STRIKING AND PERSISTENT INEQUITIES IN CHRONIC DISEASE

Chronic diseases are the leading cause of mortality and disability worldwide, and their burden is expected to continue accelerating (20, 57, 145). Chronic disease refers to conditions that last at least 1 year, require ongoing medical attention, and/or limit activities in daily life (23). Globally, 60% of deaths are attributed to four primary chronic diseases: cardiovascular disease, cancer, type 2 diabetes, and chronic lung diseases (141); low- and middle-income countries face a disproportionate burden of these deaths (57). In the United States, about 60% of adults have one chronic disease, and 40% have multiple conditions (23); about one-third of adults globally have multiple chronic conditions (23, 57, 86). These numbers will continue to rise given that many infectious diseases (e.g., HIV) are now largely regarded as chronic. Chronic diseases have significant financial consequences at the individual, societal, and health system levels as well as economic, psychological, and quality-of-life impacts on patients and families (57).

Many chronic diseases have common and potentially modifiable behavioral drivers (e.g., dietary behavior/nutrition, alcohol intake, tobacco use, physical inactivity) and related risk factors (e.g., high blood pressure; 80, 98). While these offer potential intervention points, they also are shaped by broader upstream determinants. There is growing recognition of how the social determinants of health (SDOH) and underlying structural factors and systems create and maintain avoidable, unjust, and persistent health inequities (27), including those based on race, ethnicity, and socioeconomic position (20, 26, 81, 112). SDOH are the conditions and social contexts in which people spend their daily lives (i.e., live, learn, work, play, and age) and include economic stability, education, health care access, neighborhood/built environment (e.g., housing, transportation), and social/community context (e.g., violence, discrimination, social networks; 19, 44, 93, 143, 144). Structural factors and systems include economic policies, political systems, structural and systemic racism, and societal norms. SDOH and structural determinants affect exposure and susceptibility to risk factors for chronic disease as well as disease outcomes (113).

Tobacco use, which is linked with 21 chronic diseases, offers an illustrative example. Initiation and use of tobacco products, as well as access to tobacco cessation services, are driven by personal stressors and socioeconomic factors, social network norms, and structural factors including racism—e.g., the targeted marketing of tobacco products to communities of color and the disproportionate density of tobacco retailers by neighborhood racial/ethnic composition (48, 111). Structural drivers have intersecting, multi-level effects that place historically marginalized communities at greater risk for multiple chronic diseases (27). As such, the context and complex interplay between individual-, interpersonal-, organizational-, community-, system-, and policy-level factors must be taken into account when conducting chronic disease prevention and management research.

Quantitative research has been widely used to document and describe chronic disease patterns and related inequities (71). However, because such inequities are interconnected, socially produced, and rooted in broader structures, systems, and policies (81, 112), qualitative research is critical for addressing them. Qualitative research is essential for understanding why these inequities exist (e.g., their root causes and the multi-level contextual drivers that shape inequities) as well as for informing the development and implementation of multi-sector and multi-level interventions and policies to address them, with a focus on health equity and justice.

## CONTRIBUTIONS OF QUALITATIVE RESEARCH TO FRAMING, UNDERSTANDING, AND ADDRESSING CHRONIC DISEASE INEQUITIES

**Figure 1:**
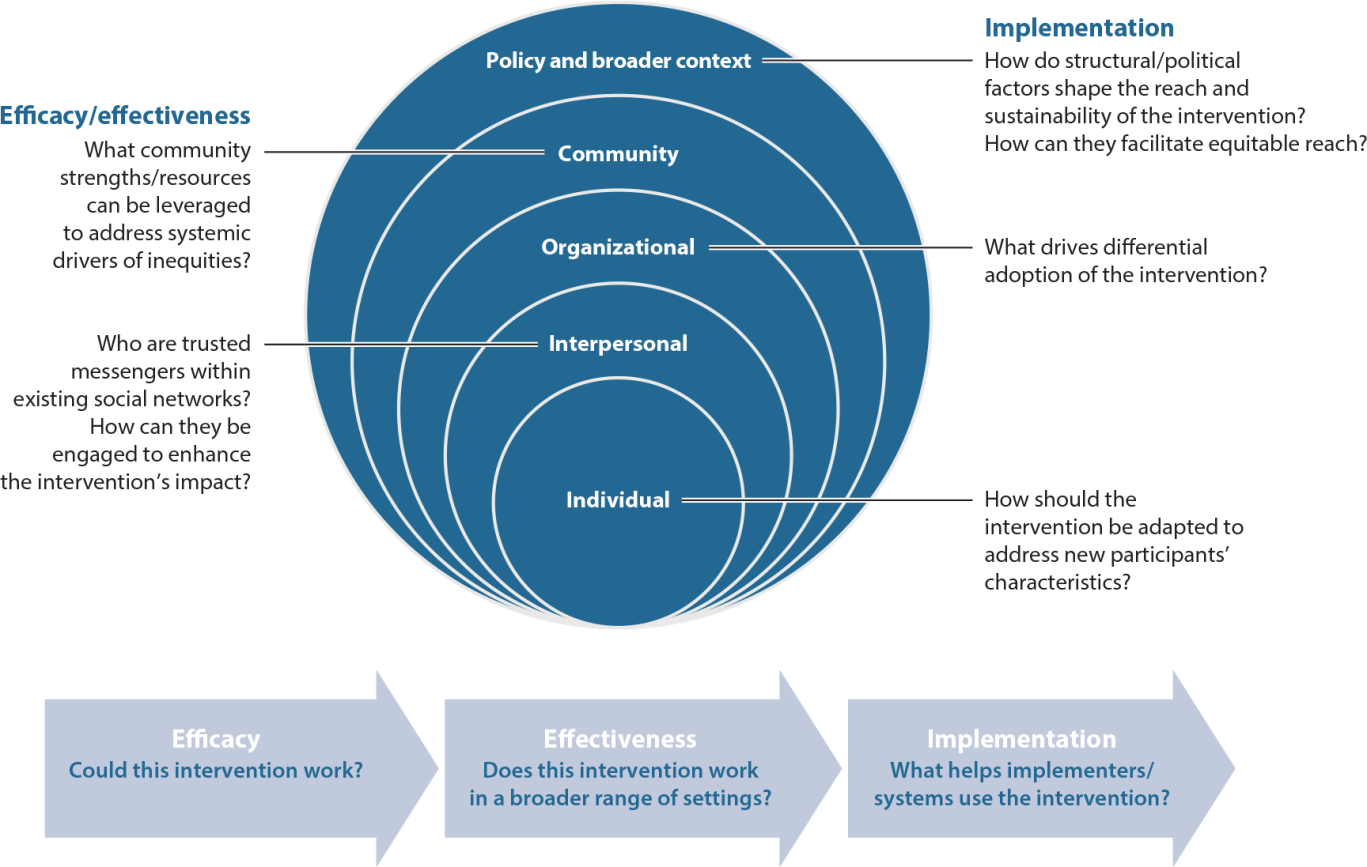
Exemplar research questions where qualitative methods can be applied to support and inform chronic disease intervention development, evaluation, and implementation to promote health equity.

The contributions of qualitative research in public health have been increasingly recognized over the past 30 years (e.g., 2, 50, 76). In contrast to quantitative research, which relies on numerical and statistical data grounded in more reductionist and positivist paradigms (e.g., a focus on hypothesis testing, objectivity, and internal validity; 65, 110), qualitative research is grounded in a constructivist paradigm. This paradigm asserts the existence of multiple socially constructed perceptions and realities versus the existence of a singular, objective truth (29, 65, 122). However, qualitative research is often selectively applied during formative research and evaluation, and its application as a method that has value across all phases of the research process is underutilized. We argue that qualitative research can play a central role in framing research questions and informing research studies related to health equity and chronic disease along the evidence generation continuum (see Figure 1 for exemplar qualitative questions that can be applied to all phases of intervention development and implementation).

Qualitative methods can play a central role in how researchers frame, understand, and identify solutions to address health inequities (53). First, these methods can provide in-depth insight into the complex real-world contexts that shape health and chronic disease patterns and deepen insights into phenomena or behaviors that remain underexplored (65). A qualitative orientation facilitates a nuanced, holistic, and contextualized understanding of the multi-level factors and processes that shape chronic disease inequities. It can help ground the data in the lived experiences of relevant communities and stakeholders (e.g., understanding patient and provider perspectives on how socioeconomic factors shape access to insulin for adults with type 1 diabetes; 126). Bowleg (16, p. 678) describes qualitative work as key to addressing differences that are “foundationally grounded in intentional, systematic, and structured discrimination based on the intersections of race, ethnicity, gender, class, sexual minority status, and ability.” Through this rich contextual grounding, qualitative methods can facilitate more justice-oriented action by advancing understanding of the mechanisms through which these inequities persist (53, 128) and by identifying differential pathways to disease diagnosis or management (e.g., 30). These insights can then be used to frame and iteratively inform or advance theories and frameworks that guide research on chronic disease inequities; they can also support the development and implementation of policies and programs to actively address them (53, 90, 91).

Additionally, by capturing and elevating the experiences of the people who are the most affected by health inequities, and often the least represented in research, qualitative data move beyond a reductionist view to capture the diversity of lived experience from multiple perspectives (e.g., patients, providers, family members, practitioners, policy makers, and community members) in their voices (65). Such methods offer the opportunity to take a systems-level perspective that incorporates individuals’ and groups’ sociocultural perspectives and lived experiences within health care systems and communities, in contrast with a narrow focus on their disease diagnosis (e.g., 24, 73). From an equity perspective, these methods can importantly support engaging with, amplifying, and centering the voices of those frequently marginalized and silenced. Such representation can also help enhance the likelihood that identified solutions are acceptable, appropriate, and sustainable within the communities they seek to serve. For example, this might include framing chronic pain as a social justice issue versus a biomedical issue and considering equity-oriented health care paradigms like trauma-informed care as relevant solutions (139).

Whereas mixed-methods designs often integrate qualitative and quantitative methods (105, 107), there is also value in stand-alone qualitative research. In the following sections, we offer (*a*) an introduction to qualitative inquiry frameworks, (*b*) common approaches to collecting and analyzing qualitative data, (*c*) considerations for ensuring and communicating rigor in qualitative research, and (*d*) recommendations for advancing work that addresses chronic disease inequities. Each section is grounded in the pursuit of improving chronic disease outcomes and health equity.

## AN OVERVIEW OF PRIMARY QUALITATIVE INQUIRY FRAMEWORKS

Researchers have identified five primary qualitative inquiry frameworks, or approaches, to inform the application of qualitative research methodologies: ethnography, grounded theory, phenomenology, narrative inquiry, and case studies (29, 88, 99) (see Table 1). Although the use of these approaches is important to ensure that qualitative research is rigorous and theory driven, they appear to be greatly underutilized in public health (76). Of note, researchers do not need to identify a singular approach and attempt to apply a strict interpretation of it. Instead, there may be value in taking a pragmatic approach and strategically integrating approaches to meet a study’s needs and goals (116).

### Ethnography

The purpose of ethnography is to understand how the culture of a given group is created, how meaning is made and understood, and how beliefs are attached to activities, including behaviors and knowledge (65, 104). Ethnographic research typically requires immersion in a cultural context (e.g., community, clinic, Internet group) and extended, ongoing observations to build rapport with community members and understand their emic (i.e., insider) perspective (29) and social organization of daily life (142). This approach is particularly useful for observing nonverbal cues and understanding issues that are sensitive or not easily measurable (32). This long-term engagement and focus on lived experiences can enhance health equity by sharing the voices of marginalized communities (11, 15, 47, 78, 92, 123–125). Researchers in public health are now also applying rapid ethnography (e.g., Rapid Assessment Procedure-Informed Clinical Ethnography) (5, 6, 109), which may be relevant in pragmatic trials, implementation studies, or settings with time or resource constraints (108).

### Grounded Theory

The goals of grounded theory are to identify the core social processes within a given social situation and to use those findings to develop a theory. This approach uses iterative data collection and analysis to inform theory development (65, 110) and can help provide a framework for further research (36). This approach is particularly useful for exploring the mechanisms and strategies for changes at the policy, community, and social levels (e.g., 133). It can help develop theory to challenge discriminatory assumptions and practices (89, 120) or contextualize health behaviors. As one example, Harley et al. (61) used a grounded theory approach to guide the development of a behavioral framework explaining the process of physical activity participation among African American women.

### Phenomenology

Phenomenology seeks to understand the essence of a lived experience and explore deeply held perceptions to investigate how the world appears to others. This requires researchers to enter into an individual’s world and suspend their preconceived notions to interpret the participant’s lived experience (29, 110). The phenomenon is the target of study (e.g., experience of stigma or cancer). In the context of health equity, understanding people’s lived experiences with a given phenomenon can enable the development of interventions to identify and address what participants perceive as sources of inequities (33, 130). For example, a phenomenological approach identified the need for social services to address intersecting issues of trauma, mental health, and violence that interfere with Aboriginal women’s chronic disease management (34).

### Narrative Inquiry

Narrative inquiry focuses on understanding lived experiences based on the stories (often temporal or episodic) of a small number of individuals who represent a case grouping of interest (29, 104, 110). The collected stories can then be examined and discussed by the researcher and the participants to explain and interpret events. As a genre particularly pertinent to illness narratives, the focus is on the diagnosis (e.g., chronic disease diagnosis) and subsequent experience (7,22,25,42). Storytelling can be a powerful tool to advance health equity research (4), as illness narratives can give voice and agency to stigmatized groups (12, 22, 43). Researchers can use life histories or life-course narratives so participants can reflect on important events in their lives and their meaning (10, 49). For example, Sprague et al. (134) charted life-course histories among women living with HIV to deepen our understanding of the nature, sequence, and timing of events shaping women’s susceptibility to HIV and incarceration.

### Case Study

A case study approach aims to gain an in-depth understanding of a single or small number of cases, entities, or groups in their real-world context at a specific time. It primarily addresses “how” and “why” questions and focuses on a defined area of action within a setting. Using this approach to gain in-depth insight into specific cases (e.g., schools with community gardens as part of obesity prevention efforts; prisons with available syringe exchange programs) can help identify potential sources of inequities within and across cases as well as identify possible targets or settings for intervention (62). For example, a case study approach was used to understand the routines and cultures that influenced health professionals’ referrals of diabetic patients to an exercise program. Using interviews, documentation, and observations that engaged providers and patients, the team was able to identify contextual supports that helped inform intervention success (87).

## COMMON QUALITATIVE DATA COLLECTION METHODS

The frameworks described above can be used with a variety of qualitative data collection methods, including in-depth and unstructured interviews, focus groups, participant observation, document/archival review, and participatory methods and engagement (29, 88, 99). Table 2 offers an overview of these methods and highlights some strengths and weaknesses to consider in their application. Decisions around which methods to use are shaped by the research framework, research question, intended products or outcomes, and feasibility (103).

### Interviews

Interviews are a common method of qualitative data collection (76). There are four primary types of interviews: (*a*) closed, fixed-response interviews, where questions and response categories are predetermined; (*b*) standardized, open-ended interviews, where the wording and sequence of questions are predetermined but the questions are open-ended; (*c*) semi-structured interviews, where the topics are specified but the interviewer decides the sequence/wording during the interview and can explore new topics of interest; and (*d*) unstructured interviews, where the questions emerge during the interview itself based on the context. Interviews can occur in person, over the phone, or over videoconferencing. Interviewing is typically appropriate for sensitive or stigmatized topics because it occurs one-on-one and is confidential; it can also be useful when interviewing experts on a subject. However, it necessitates researchers who are knowledgeable about the subject matter, sensitive to the sociocultural context, and able to accommodate lengthy time investments (29, 76).

### Focus Groups

This method employs a group discussion with the goal of assessing social norms and group interactions around a topic in order to produce data and insights that would be hard to obtain otherwise. Focus groups usually have 8–10 participants, which may require recruiting more than twice that number of participants (77). Focus groups are commonly used to generate feedback on study materials, programs, or public health innovations. Focus groups are useful to reflect shared experiences or social norms (e.g., when participants are similar); they can also be useful for engaging stakeholders (58). Because confidentiality cannot be guaranteed, focus groups should not be used to examine sensitive behaviors, and it can be difficult to manage participants and dynamics. The unit of analysis is the group itself (not the participants within the group); 6–8 focus groups are often required to adequately represent the range of experiences on a topic. When generating focus groups, researchers should try to ensure that groups have shared experiences or commonalities (e.g., by age, disease history, neighborhood, or insurance coverage) and should also typically avoid combining individuals with differing levels of power (e.g., providers and patients).

### Participant Observation

Participation observation includes interacting with and observing people during their daily lives to reveal unspoken aspects of social context (29). Observation enables examination of what people actually do (versus what they say they do). This is particularly useful for studying stigmatized conditions (e.g., substance use) where the systematic observation of people as they engage with care providers, family members, and people with whom they use drugs can shed light on barriers or facilitators that would go unmentioned in an interview, either because people do not perceive them or because they seem unchangeable or stigmatized. Participant observation can help us understand behaviors, perceptions, phenomena, and the contextual factors that impact behavior or implementation across stakeholders.

### Document/Archival Review

Document or archival reviews involve the retrieval and analysis of documents, archives, and texts intended as aspects of material culture (i.e., physical objects and practices that constitute people’s environments). Content analysis of these texts and documents is useful for understanding institutionalized forms of knowledge and information as well as the context and history of a given phenomenon. Examples include fieldnotes (e.g., 70), periodic reflections (38), diaries or other annotated logs of observed processes (e.g., 21, 28, 115), and materials, objects, and items used to decorate physical spaces. For example, materials such as posters, toys, television programming, and pamphlets at HIV clinics can signal forms of inclusion/exclusion to patients based on age, sexual, or gender identity (136).

### Participatory Methods and Engagement

Participatory activities involve engaging participants in knowledge production around an issue of interest (96). Exemplar participatory methods include the creation of hand-drawn maps of a community (e.g., showing where people spend time), body mapping, photovoice, and life history drawings (67). These participatory methods engage participants to create data that represent their own health and well-being. In this way, participants are cocreators of knowledge and engage in the dissemination and translation of the work they produce. There are certain types of meaning or experiences that can be challenging to put into words, particularly for marginalized groups or youth. The strength of participatory methods is that they place the power in the hands of participants as opposed to researchers and allow participants to shape how meaning is communicated. For example, photovoice allows participants to document their daily lives and interactions in the communities and with systems using cameras. They reflect on community strengths and challenges, and the sharing of photos (and now videos) using group discussions, critical dialogues, and even public art exhibitions can reach a diverse range of audiences, including policy makers and institutional leaders. The goal is to support both knowledge creation and action, that is, community change through policy change (140).

### Summary

Importantly, qualitative methods are unique in that they provide greater opportunities to level or shift the power dynamic from the researcher to the participant. From an equity perspective, it is critical to consider and ask which methods may be preferable to facilitate the engagement of groups that face health or social inequities.

## CONSIDERATIONS FOR SAMPLING IN QUALITATIVE RESEARCH

Overall, qualitative research typically has smaller sample sizes than quantitative research; the needs of a given study are a function of the research goals, inquiry framework, and data collection methods (18). Data saturation, the point at which additional data collection and analysis cease providing new insights (102), is integral to many qualitative sampling plans and often reflects the priority placed on depth of inquiry and ability to assess the nuance of individuals’ lived experiences (18). A recent review found that studies reached saturation at relatively small sample sizes (9–17 interviews, 4–8 focus group discussions), especially for studies with relatively homogenous study populations and narrower objectives (66). Another useful way to consider sampling requirements is to examine information power. This concept describes how sample size may be determined by a combination of study aim(s), sample specificity, theoretical framework, quality of data collection, and analytic approach (84).

Qualitative research relies on numerous sampling strategies, many of which are purposive. Here, participants are chosen because they represent certain groups, individuals, or perspectives and can speak directly to the research question (106); in some cases, sampling may be stratified to represent diversity of experience by race/ethnicity, socioeconomic position, or other characteristics (30). Other strategies may involve more theoretically informed sampling, which is guided by ongoing data collection and refinement of theory. For example, deductive theoretical sampling could be applied a priori to deepen or verify specific theoretical constructs among a given population (e.g., discrimination among patients with HIV); by contrast, inductive grounded theoretical sampling would involve determining the sample as the theory develops from exploratory to verification (110). Snowball sampling, in which participants suggest the subsequent participants, may be necessary when the full range of relevant individuals may not be reachable by researchers at the outset.

To enhance equity, it is essential that researchers be transparent and intentional in developing their sampling plan, as this has implications for whose perspectives are obtained and reflected in the research, whose voices are heard, and who is excluded. For example, researchers may want to learn about people living with kidney disease, but if they only sample people who attend a clinic they may not learn about all relevant barriers to care because they will miss those who cannot make it to the clinic.

## STRATEGIES AND CONSIDERATIONS FOR QUALITATIVE DATA ANALYSIS

There are a diversity of strategies used in qualitative analysis (8, 94, 121, 122, 135); these are guided by the research question, nature of data, and application of inquiry frameworks (29, 110, 122). The limited word counts in many public health journals prevent sufficient data sharing regarding analytic approaches, particularly related to data synthesis and summary as well as to the identification of patterns and interpretations. However, such transparency is imperative, as it can enhance opportunities for replication and improves the rigor of qualitative research (95). The use of supplemental materials, where allowed by the journal, can offer the necessary detail to interested readers.

Coding is a foundational analysis strategy for qualitative research. Typically, coding breaks down the text into units (e.g., blocks with a particular meaning) in order to describe and label the data. Codes are then applied to all transcripts and examined to identify categories of codes and, later, themes of interest. Coding can include either inductive or deductive orientations, or a combination of the two (117). An inductive coding orientation would involve reviewing the data to develop initial codes or patterns, including the development of new theory, whereas a deductive coding orientation would involve developing an initial codebook based on the research questions, the interview guide, existing theoretical or conceptual frameworks, or a literature review (65).

Thematic and content analysis are common approaches for analyzing qualitative textual data or narrative materials. For content analyses, researchers take a systematic coding approach to examine large amounts of textual data, with the goal of identifying patterns in words (who says what), their relationships, structures, and impacts (52, 114). In contrast, thematic analysis has been defined as “a method for identifying, analyzing and reporting patterns (themes) within data” (17, p. 79). Other strategies include the use of matrices to identify patterns, themes, and relationships during the analytic process (95, 122). Quotes are commonly used to report key findings from synthesized data. Qualitative data management software, including ATLAS.ti, NVivo, NUD-IST, Ethno-graph, and Dedoose, can facilitate data organization, data coding and retrieval, and summary code reports.

From an equity perspective, some of the ways we center quality and rigor involve returning data and data summaries to the community to confirm that the researchers’ interpretations of the lived experiences of groups are adequately reflected (e.g., through member checking); such activities can also facilitate engagement and can promote inclusion.

## VALIDITY, RELIABILITY, AND REFLEXIVITY IN QUALITATIVE RESEARCH

Quantitative researchers may question the rigor of qualitative work because they do not fully understand its underlying framework and systematic activities. Tracy proposed a flexible set of practices that can serve as markers of quality in qualitative research (137). We build on that set here and emphasize applications for considering equity.

1. Importance of the topic. The topic must be relevant and worth the investment of time by researchers, participants, and relevant stakeholders. From an equity standpoint, this may include details of how the topic aligns with the priorities or concerns of the intended communities and systems that could utilize the findings.
2. Rigor. Rigor indicates that the study employs theoretical constructs and provides sufficient detail and rationale for choosing a given construct. Data collection and analytic processes are clearly described, and the investigators use audit trails (i.e., maintaining records of all steps in the research process). The samples and context of the study are clearly described, along with details of the duration and intensity of engagement with the study setting (29).
3. Reflexivity. Qualitative studies should offer details about the positionality of the researcher(s) in relation to the study participants and the context in which the research occurs. Reflexivity recognizes the ways in which that positionality may influence the research process (29, 110). Relevant details may include research team members’ personal or professional characteristics in the context of a given study’s participants and settings (e.g., gender, age, race/ethnicity, geography). Considerations of positionality are particularly important for addressing equity, given the ways in which institutions and power structures shape inequities and may silence the voices or perspectives of groups that have been historically marginalized or excluded. An equity lens on reflexivity also prompts the thoughtful composition of research teams to include individuals with lived experience that is relevant to the issue at hand.
4. Reliability. Investigators should utilize a range of methods to enrich and support their findings and enhance reliability. For example, it may be useful to apply member reflections or peer debriefing and to share early findings with participants or individuals familiar with the topic (i.e., member checking). This would then drive iteration of the analysis (29). Another example is to use triangulation, which explores consistencies and inconsistencies of findings generated using different methods, researchers, data sources, and theories (39). Additionally, the study can explore negative or contradictory cases and refine the analysis accordingly (29).
5. Importance of the findings. The resulting contribution should be meaningful and framed in terms of utility and transferability. This marker of quality is particularly important given the urgency of prioritizing action for addressing health equity in public health.
6. Ethics. The research should be conducted so as to meet not only the minimum standards of human subjects research but also broader ethical conventions regarding the context and participants, the relationships between individuals and institutions, and the potential for the findings to impact health equitably. The last piece emphasizes the role of translation and includes important questions about what happens after data collection: With whom are they shared, and how? To what end?
7. Coherence. Coherence indicates that the study uses methods and procedures that match the project goals and underlying philosophical assumptions. The research topic, findings, and interpretations are clearly connected and placed in the context of the public health literature.

Another recommendation when communicating qualitative findings is to consider them in terms of four types of validity, as described by Johnson (74). First, descriptive validity emphasizes accuracy in reporting and can be demonstrated through the use of transcripts, multiple observers, and real-time documentation. Second, interpretive validity examines the accuracy of the interpretive process. Member reflections can bolster this, as can the use of direct quotes or data from other sources. Third, theoretical validity asks whether the explanation fits the data. Bringing readers along through the process by demonstrating how conclusions were reached as well as the depth of knowledge among the investigators is key. It is also possible to attempt to use different theories and peer feedback to seek alternate explanations that may enrich the results. Finally, internal validity refers to the extent to which researchers can claim causal relationships. This requires attention to other potential explanations and consideration of the depth and breadth of the data that support such claims.

Researchers can integrate findings across qualitative studies through synthesis processes. This approach may include conducting a comprehensive review or using iterative processes to explore data to build or extend theory. Common methods for pooling qualitative evidence include thematic synthesis, narrative and meta-narrative approaches, meta-ethnography, cross-case analysis, and realist review (37, 60).

## APPLYING QUALITATIVE METHODS TO PROMOTE EQUITY AND ADDRESS CHRONIC DISEASE INEQUITIES

In the sidebar titled Summary and Explication of the Advantages of Qualitative Methods for Understanding and Addressing Health Inequities, we summarize key opportunities for applying qualitative methods to advance health equity research, and in the subsections below we conclude by highlighting priority areas for applying qualitative methods that hold great promise for addressing chronic disease inequities.

### Qualitative Research Can Advance, Inform, and Refine Theories Around Equity, Social Determinants of Health, Racism, and Power in the Context of Chronic Disease

Qualitative methods are ideal for critically examining understudied and complex structural and root causes of health and disease, including racism, power, and other social, structural, and political determinants of chronic disease inequities (53, 63). Future research should leverage and apply the qualitative inquiry frameworks described here to inform and refine equity-relevant conceptual theories, frameworks, and models. To cite one example, Sprague et al. (133) used a grounded theory approach to describe the impact of SDOH on African Americans living with HIV in Mississippi. Researchers developed new social theory from the data that identified how SDOH and structural factors—including the legacy of slavery, the political economy of the US South, conservative political and social attitudes, stigma, and mass incarceration—shaped individuals’ health. Such data highlight the experiences of socially marginalized communities and can inform social science understandings of the complex interrelationships between factors and the processes through which inequities are created, are reinforced, and shape chronic diseases (53).

We recommend a greater application of qualitative research to test and refine theories that examine the mechanisms through which SDOH, racism, and power imbalances create health inequities. For example, qualitative methods could be informed by or could apply, test, and refine theories that center health equity or racial equity, including Public Health Critical Race Praxis (40, 41) or theories of structural violence (128, 132). As Bowleg (16) noted, qualitative methods are not inherently progressive unless they are grounded in theory and epistemology in ways that shape and inform how they address health equity. Whether explicitly named or not, there is always an underlying theory or framework that undergirds the research (65). Given its constructivist orientation, qualitative research is well suited to take a critical stance and help reflect on and inform the types of questions we ask and who is invited to share their expertise (54). In this way, we can move away from a shortcoming in the current health promotion evidence base—i.e., the fact that much of the research evidence emphasizes single-level action, even though we know that multi-level action is much more likely to create sustainable and equitable impacts on health (138).

### Qualitative Methods Can Be Applied to Address Inequities Across Both the Chronic Disease Continuum and the Evidence Generation Continuum

Qualitative research provides insight into different phases of the chronic disease evidence generation continuum. This includes a contextualized understanding of the factors that place people at risk for disease, the conditions affecting access to screening and care, patient experiences during acute treatment, and challenges to chronic disease self-management (14, 51, 56, 59, 82, 129). Qualitative research can include longitudinal and prospective explorations across multiple time points along the chronic disease continuum to highlight places for intervention or for improving the delivery of care for patients across the disease trajectory (e.g., 69, 119). The majority of chronic disease research has limited the use of qualitative methods to informing formative research and to understanding factors that affect the risk of, or experiences with, specific aspects of chronic disease care or treatment. Qualitative work has also been applied to inform the design or development of interventions or clinical care delivery (e.g., 100, 118) and, more recently, the design of trials (13).

As a complement, we also encourage researchers to apply qualitative research methods across the translational research continuum with a focus on promoting equity (Figure 1). Although they are underutilized in the evaluations of interventions, including within randomized controlled trials, qualitative methods hold great value by providing us with a better understanding of why interventions succeed or fail (e.g., 9) and of which interventions work for which populations/settings. This is particularly necessary in order to understand the potential exacerbation of inequities and the mechanisms through which complex interventions operate (e.g., 35, 131).

As several publications describe (e.g., 58, 101), implementation research places greater value on qualitative methods, including ethnographical approaches (46). Such research can help elucidate the how and why of implementation successes and failures in complex, dynamic real-world contexts and from the perspectives of a range of stakeholders (e.g., patients, providers, administrators, policy makers). It can shed light on contextual and organizational barriers and facilitators to adoption, implementation, and sustainability, and it can inform the development, delivery, and examination of strategies to actively promote implementation. To advance health equity, we recommend that researchers apply qualitative methods and frameworks in implementation research with a more theoretically critical perspective. This includes an examination of reflexivity in knowledge production (e.g., whose evidence and expertise are valued); an integration of theories related to intersectionality, racism, power, and structural violence; and a consideration of policies, governance, and societal factors that shape health equity (127, 132).

### A Broader Range of Qualitative Methods Can Be Applied, Including Participatory Approaches, to Promote Health Equity

The full range of qualitative research methods has not been leveraged in public health and health care, particularly in chronic disease research. We encourage the use of diverse qualitative methods to inform the broad range of research questions relevant to chronic disease and inequities. To cite one example, Davis et al. (31) offer a range of innovative methods, such as diaries, spiral walks, and community mapping, to complement randomized controlled trials evaluating complex health interventions. From a health equity perspective, there is particular value in applying underutilized participatory qualitative methods to facilitate community capacity building, engagement, and data ownership (1). For example, a participatory community health assessment used interviews and focus groups with community partners in a Mexican immigrant neighborhood, involving community members in a participatory analysis (64). The findings included responses to a community-administered health survey and an oral history component that provided insight into the community’s needs. Such participatory qualitative methods provide a more comprehensive understanding of the determinants of health inequities (75) and have the potential to engage and collectively activate socially marginalized groups to collectively build knowledge for action to improve health.

Photovoice is the most commonly used participatory method to provide insight into a community’s assets and people’s daily experiences, including in the context of chronic disease (e.g., 3, 68, 79). We encourage the continued application of a range of participatory methods to qualitative research to center the voices of key stakeholders (e.g., 72, 97). Such participatory visual qualitative methodologies (45), including body mapping and community mapping, have been applied to better understand the complex causes of health inequities (55, 83). Visual participatory methods can also be applied in the context of understanding and evaluating the findings of interventions and trials (85), and they may provide important insights into promising avenues for promoting equity, including for stigmatized populations or health issues.

## CONCLUSION

Qualitative research methods can prioritize a health equity lens that shifts the focus away from individualistic biological, genetic, and psychosocial explanations of inequities. Instead, this method argues for a more in-depth, theoretically informed understanding of the complex and interacting sociopolitical and cultural contexts, norms, and structures that shape health and disease, and it informs actionable solutions to address them. Within this frame, the value of qualitative methods is clear: They are critical for providing a contextualized understanding of how inequities are created and maintained from a multi-level perspective as well as analyzing details about intervention and implementation successes and failures. Through stakeholder-engaged qualitative research, with the explicit involvement and engagement of the communities that experience inequities, as well as a consideration of the actors within systems that produce or maintain these inequities, we can identify actionable intervention points and policies to address chronic disease inequities.

## Figures and Tables

**Table 1:** Characteristics of selected inquiry frameworks in qualitative research

Approach	Goal	Disciplinary background	Sampling approach and sample sizes	Common types of data	Analysis approaches	Research question framing
Ethnography	To examine the culture of a community (e.g., city, business, family)	Anthropology, sociology	Purposive sampling; can use multiple interviews with the same 15–20 people and one-off interviews (*n* = 50–60)	In-depth interviews, unstructured interviews, participant observation, textual and content analysis, archival research	Applying open and theoretical coding approaches to generate themes about the community of interest	To gain a cultural understanding
Grounded theory	To develop a theory based on collected data	Sociology	Theoretical sampling (*n* = 20–60)	In-depth interviews	Using open, axial, and selective coding to generate theory about the topic of interest	To generate theory
Phenomenology	To understand the lived experiences and perspectives of participants	Nursing, education, philosophy, psychology	Individuals with lived experience; multiple interviews with 8–10 people	In-depth interviews, art and poetry, memos, participant observation	Focusing on textual and structural description and on significant statements that demonstrate the essence of a person’s lived experience	To seek meaning
Narrative inquiry	To explore an individual’s life through their stories	Anthropology, literature, history, sociology, psychology	Individual(s) with direct illness experience (usually 1–2 people)	In-depth interviews, documents (e.g., diaries)	Examining data to uncover stories, develop themes, and identify breaks in the narratives	To tell a story
Case study	To investigate the development of a single event, place, or situation (e.g., school, hospital)	Psychology, law, political science, medicine	Study of 1–2 cases; approximately 20–50 people sampled within a case	In-depth interviews, participant observation, textual and content analysis, artifacts	Applying coding to develop themes within and across cases	To explore a given example in depth

**Table 2:** Characteristics of commonly applied qualitative data collection methods

Approach	Characteristics	Strengths	Weaknesses
Semi-structured in-depth interviews	Topics are predetermined, questions are written out, and all interviewees are asked similar questions, with the opportunity for the researchers to ask additional questions. Interviews are one-on-one.	Having an outline increases comprehensiveness of the data; interviews remain relatively conversational.	There is less flexibility to tailor the interview topic to a specific individual or setting; topics important to some people may be difficult to access through a structured approach.
Unstructured interviews	Questions emerge from the context and are not predetermined; interviewer decides sequence and wording of questions during interview. Interviews are one-on-one.	Interviews are adapted to a specific individual and context; this increases salience of questions and provides insider perspectives.	Different information is collected from each person; data can be less systematic or comprehensive.
Focus groups	Topics are predetermined and questions are written out, though the moderator can update as needed. There are often 8–10 individuals per focus group.	Focus groups allow researchers to assess knowledge and attitudes as well as how people in a group think about a given topic; they provide opportunities for participants to build on others’ ideas.	There is less in-depth information about a given topic; groups may be challenging to manage, particularly in the presence of a dominant speaker; groups may not work for sensitive content.
Participant observation	The researcher engages in the setting and can observe either passively (from afar) or actively (engaging in activities with those being observed).	Participant observation allows researchers to observe people as they behave rather than how they say they behave; researchers can gain understandings of context to inform other methodological approaches.	The interviewer must incorporate their own reflexivity and understand how that shapes their experiences; people may change behavior in the presence of the researcher.
Document/archival reviews	This method is based on the analysis of existing materials (e.g., diaries, pamphlets, health campaigns, artwork).	Content analysis incorporates materials that affect how people understand their experiences and allows researchers to examine how people express themselves outside of interviews.	This method is not always available depending on location or individual; systematic comparisons may be difficult.
Participatory methods and engagement	These are methods that engage, and are driven by, the participants themselves (e.g., photovoice, body mapping, community mapping).	These methods allow participants to shape the research question in ways that are meaningful to them; participants who may struggle to verbalize an idea can participate.	These methods can be time consuming and expensive; they can privilege some groups and mask power differentials; the researcher typically conducts the analyses; the results may not be accepted by some audiences.

## SUMMARY AND EXPLICATION OF THE ADVANTAGES OF QUALITATIVE METHODS FOR UNDERSTANDING AND ADDRESSING HEALTH INEQUITIES

Some examples of the advantages of using qualitative methods in the context of health equity research are listed below.

- Qualitative methods can provide a rich, holistic, and nuanced understanding of contextual factors and root causes that shape health inequities, particularly at the policy, societal, community, and social contextual levels, and especially where quantitative measures may be limited or fail to fully capture important issues.
- Qualitative methods support diverse teams of researchers (e.g., those with formal research or practice credentials, community members, and other stakeholder experts) gain a more in-depth understanding of the complex and dynamic mechanisms, behaviors, and processes through which inequities are created and reinforced, and they can inform the development and refinement of relevant theories, models, and frameworks.
- Qualitative methods amplify and center the voices of communities that experience social and health inequities, helping researchers and policy makers understand their experiences and co-create or develop relevant and grounded solutions.
- Qualitative methods can capture the multiple realities and perspectives of stakeholders who have divergent notions of why health inequities exist or which interventions are feasible and appropriate to implement.
- Qualitative methods are especially useful when a phenomenon is new or there has been little previous research in an area or in partnership with a population/setting.
- When quantitative measures are not available or have not been psychometrically validated among the population of interest, qualitative methods could inform the development of measures that are culturally and contextually appropriate.
- Qualitative methods allow researchers to understand how and why an intervention, policy, or implementation strategy works for a specific population, so it can be replicated and scaled to target chronic disease inequities.
- Conversely, when an intervention or implementation strategy was null or ineffective for a particular community or setting, qualitative methods may be needed to understand why it did not work.
